# Fabrication of a Bare Optical Fiber-Based Biosensor

**DOI:** 10.3390/mi10080522

**Published:** 2019-08-08

**Authors:** Yu-Jun Zhang, Jin-Cherng Hsu, Jia-Huey Tsao, Yung-Shin Sun

**Affiliations:** 1 Department of Physics, Fu-Jen Catholic University, New Taipei City 24205, Taiwan; 2 Industrial Technology Research Institute, Hsinchu 31040, Taiwan

**Keywords:** optical fiber sensor, surface plasmon resonance, biosensor, binding kinetics

## Abstract

A bare optical fiber-based biosensor is proposed for measuring the refractive index of different liquids and the binding kinetics of biomolecules to the sensor surface. This optical fiber sensor is based on the Kretschmann’s configuration to attain total internal reflection (TIR) for surface plasmon resonance (SPR) excitation. One end of the bare optical fiber is coated with a gold film. By guiding the light source from the other end into the optical fiber, the light is reflected from the gold-deposited end and the surface evanescent wave is excited in the gold film-transparent material interface. Methanol and ethanol solutions with different refractive indices are used for measuring the corresponding changes in the peak values of the spectra and calculating the corresponding sensitivities. These values are experimentally determined to be in the order of 10^−4^~10^−5^ refractive index unit (RIU). Binding of proteins onto the sensor surface is also monitored in real time to obtain the binding kinetics. We believe that, in the future, this optical fiber sensor can serve as a useful biosensor for in situ measurement of allergens, antibody–antigen interactions, and even circulating tumor cells in the blood.

## 1. Introduction

Surface plasmon resonance (SPR) refers to the consequence of exciting a surface-bound electromagnetic wave at the metal–transparent material interface. Such a phenomenon occurs at a certain incident angle (with wavelength fixed) or a certain wavelength (with incident angle fixed), and its signal is related to the dielectric constants of the metal, the transparent material, and the ambience. This technique has long been applied to the design of biosensors, mainly because it provides label-free and real-time characterization of biomolecules [1,2]. Fluorescence-based detection methods have been routinely used in bio-laboratories because they have excellent sensitivity and a large selection of labeling agents. However, fluorescently labeling probes can significantly change their binding affinities to corresponding targets [3]. Therefore, SPR-based biosensors have drawn increasing attention recently because the biomolecules under investigation can remain intact. To attain SPR, a prism is required to set up the Kretschmann’s configuration under total internal reflection (TIR) [1]. As an alternative, surface gratings can be used to diffract light to various angles with at least one inducing SPR [4]. In these two settings, bulky prisms or complicated surface modifications are required, limiting their in vitro applications.

Optical fibers, having the advantages of small size, low cost, and flexibility, provide another platform for attaining TIR, and thus SPR. A large number of SPR-based optical fiber sensors (OFSs) have been reported in measurements of temperature, refractive index, pressure, and even biomolecules [2,5]. In 1990, Villuendas and Pelayo reported one of the very first SPR-based OFSs, where the sensitivity and dynamic range in detecting aqueous sucrose solutions were experimentally determined to be 3.5 × 10^−6^ RIU and 3.5 × 10^−3^ RIU, respectively [6]. Recently, D-shaped and D-type OFSs have been developed due to their easy fabrication and high sensitivity. In a D-shaped OFS, the cladding is partially removed, while in a D-type OFS, the whole cladding and a portion of the core are removed. The polished, flat surface, serving as the sensing area, is then coated with a metal (usually Au) film to attain SPR. Luan et al. presented a D-shaped hollow core micro-structured optical fiber (MOF)-based SPR sensor where the analyte was deposited directly onto the D-shaped flat surface instead of filling the fiber holes [7]. The corresponding maximum phase sensitivity was numerically determined to be 50,300 deg/RIU/cm [7]. Wang et al. proposed a D-type OFS based on Kretschmann’s configuration, showing a sensitivity of 2 × 10^−4^ RIU [8]. Recently, a curved D-shaped OFS was designed and fabricated for sensing changes in the refractive indices of liquids. It was found that the fabrication parameters including the curvature of the fiber, the unpolished depth of the fiber core, the thickness of the deposited gold films, and the incident angle of light upon the gold-deposited surface affected the sensitivity of this sensor [5]. This OFS could be combined with a microfluidic chip for reducing sample consumption in biological applications [5].

Although the above-mentioned SPR-based OFSs have relatively high sensitivities, complicated fabrication processes such as peeling, embedding, and polishing are required in most of them. Moreover, the sensing area of these OFSs is located in the middle of or inside the optical fiber, making practical applications difficult. To overcome these drawbacks, a bare optical fiber-based biosensor is proposed in this paper. This OFS is based on the Kretschmann’s configuration to attain TIR for SPR excitation. One end of this bare optical fiber is coated with a gold film of about 40 nm in thickness. When the light source is guided from the other end into the optical fiber, it is reflected from the gold-deposited end and the surface evanescent wave is excited in the gold film–transparent material interface. As the refractive index of the ambience next to the gold film changes, the peak value of the reflection spectrum shifts accordingly. The present OFS shows a sensitivity in the order of 10^−4^~10^−5^ RIU for measuring liquid samples. Binding of proteins to the sensor surface is also recorded in real time to obtain the reaction kinetics.

## 2. Materials and Methods

### 2.1. SPR Phenomenon

**Figure 1:**
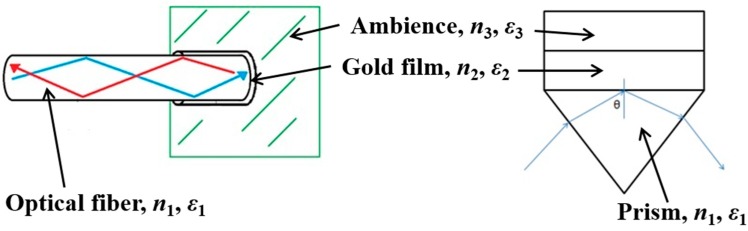
The present optical fiber sensor (OFS) (**left**) resembles the Kretschmann’s configuration (**right**).

As shown in Figure 1, the present OFS (left) resembles the Kretschmann’s configuration (right). TIR is attained inside the bare optical fiber, similar to that inside a convenient prism. At a certain incident angle (with wavelength fixed) or a certain wavelength (with incident angle fixed), the evanescent wave propagating along the metal–transparent material interface can interact with the plasma waves on the surface, excite the plasmons, and cause resonance. This resonance occurs when the following relation is satisfied (*θ* is the incident angle; *ε*_1_, *ε*_2_, and *ε*_3_ are the optical dielectric constants of the fiber/prism, the gold film, and the ambience, respectively):

(1) $$\sin\theta =\sqrt{\frac{\left|\varepsilon_{2}\right|\varepsilon_{3}}{\left(\left|\varepsilon_{2}\right|-\varepsilon_{3}\right)\varepsilon_{1}}}\;.$$

In addition, the penetration depth *d* of this surface evanescent wave is defined as (*λ* is the wavelength of the incident light; *n*_1_ and *n*_3_ are the refractive indices of the fiber/prism and the ambience, respectively):

(2) d=λ2πn12sin2θ−n32 .

As the refractive index of the ambience changes, the SPR condition, Equation (1), and the penetration depth, Equation (2), change accordingly, resulting in shifts in the peak value of the reflection spectrum.

### 2.2. Fabrication of the OFS

**Figure 2:**
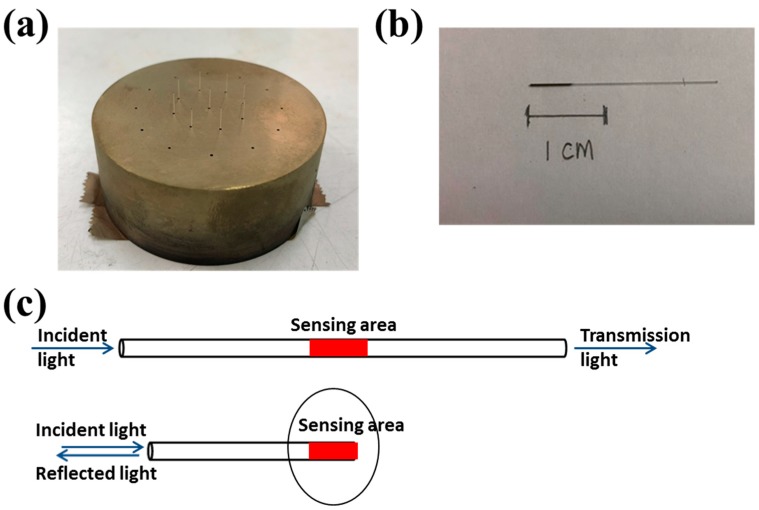
(**a**) The customized sample holder for Au sputtering, (**b**) the bare optical fiber coated with the Au film, (**c**) the sensing areas in most of the D-shaped and D-type OFSs (top) and the present OFS (bottom).

Multi-mode bare optical fibers (with core only and flat end face) with a diameter of 250 μm and a length of 2.5 cm were used in this study (Taiwan Fiber Optics Inc., Taipei, Taiwan). First, the optical fiber was cleaned with isopropyl alcohol (IPA) for 5 min in an ultrasonic cleaning machine. After being dried inside a fume hood for 5 min, it was inserted into one hole of the customized sample holder (see Figure 2a). This holder was designed so that only a length of 5 mm of the fiber was subject to Au coating. Before the DC sputtering, the chamber was vacuumed to a background pressure of about 1 × 10^−5^ torr. Then Ar gas was flowed into the chamber at a rate of 60 sccm to attain a working pressure of 5 × 10^−3^ torr. With a DC power of 20 W and the sample holder rotating at 30 rpm, the optical fiber was coated with an Au film of about 40 nm (see Figure 2b). This thickness was verified ex situ with an ellipsometer (VASE M-2000U, J. A. Woollam Company, Lincoln, NE, USA). As shown in Figure 2c (top), in most of the D-shaped and D-type OFSs, the sensing area is located in the middle of the fiber [5,9,10]. In the present OFS (see Figure 2c, bottom), the sensing area is at one end of the fiber, making it suitable for in situ and clinical applications such as direct insertion into blood vessels with a syringe.

### 2.3. Materials

Methanol and ethanol solutions with different concentrations were prepared to have a range of refractive indices. These refractive indices were calculated [11,12] and further checked with an Abbe refractometer (N.O.W., Tokyo, Japan). For example, for methanol solutions, the refractive index was calculated as:

(3) $$n_{solution}=n_{water}+0.01738\times \left(\frac{V_{methanol}}{V_{solution}}\right).$$

In this equation, *n_solution_* and *n_water_* are the refractive indices of methanol solution and water, respectively, and *V_methnaol_*/*V_solution_* is the volume ratio of methanol to methanol solution. With *n_water_* = 1.33, solutions having *n_solution_* = 1.3354, 1.3360, 1.3368, 1.3383, and 1.3412 were obtained by changing *V_methnaol_*/*V_solution_*. These values were further measured to be 1.3364, 1.3370, 1.3378, 1.3391, and 1.3402. Errors were less than 0.07%.

Bio-samples N-Hydroxy-succinimidy-propionate octa(ethylene glycol)-disulfide(C_46_H_80_N_2_O_24_S_2_, M.W. = 1109 Da) and bovine serum albumin (BSA, M.W. = 66.5 kDa) were purchased from Sigma and diluted to desired concentrations in 1× phosphate buffered saline (PBS) buffer.

### 2.4. Experimental Setup

**Figure 3:**
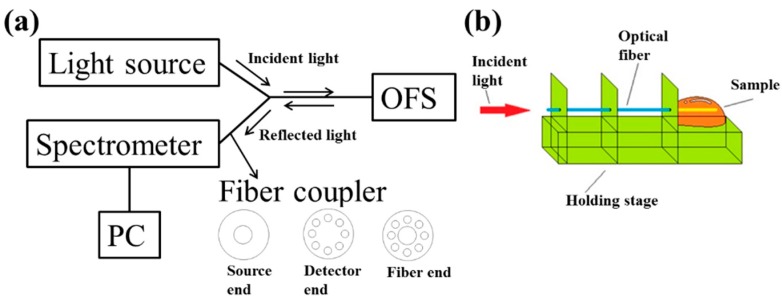
(**a**) The experimental setup, (**b**) he customized fiber-holding stage.

As shown in Figure 3a, the experimental setup contained a light source, a fiber coupler, the OFS, and a spectrometer. A tungsten halogen lamp (SL1, StellarNet Inc., Tampa, FL, USA), with a spectrum between 400 nm and 900 nm, was connected to one end of the Y-shaped fiber coupler. After being reflected from the OFS, the light was collected by a spectrometer (USB2000+XR, Ocean Optics, Largo, FL, USA) connected to the other end of the coupler. This spectrometer has a scanning range from 400 nm to 900 nm and a resolution of 0.3 nm. The fiber coupler is designed to separate the light from the source from that of the detector (see Figure 3a). Prior to experiments, the light source was warmed up for about 30 min, and the OFS was mounted onto the customized holding stage shown in Figure 3b. Twenty μL of the liquid sample was pipetted onto the sensing area, and a stable spectrum was recorded after a waiting time of 10 min. Before the next measurement, the sensing area of the fiber was cleaned with a lens tissue, 20 μL of 1× PBS, and then with a lens tissue again. For specific types of solutions, the spectra were recorded from the smallest refractive index to the largest one, and three independent experiments were performed to obtain the standard error of the mean (SEM).

To effectively capture proteins, the Au-coated optical fiber was first reacted with the disulfide polymer at a concentration of 1 mM for 30 min. After clearing, 20 μL of BSA at a concentration of 3 μM was pipetted onto the sensing area, and the spectra were recorded every 5 min for 1 h.

## 3. Results and Discussion

### 3.1. Spectra

**Figure 4:**
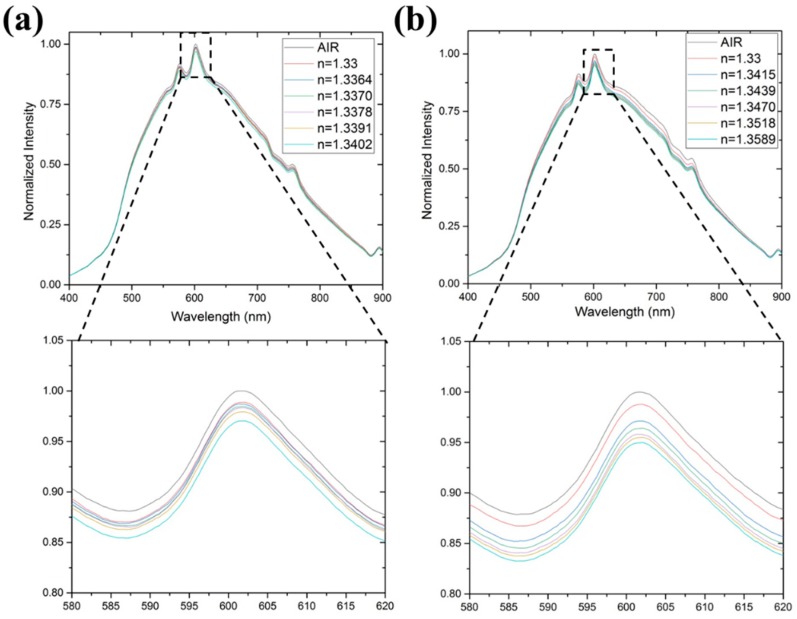
The normalized spectra of (**a**) methanol and (**b**) ethanol solutions with different refractive indices.

Figure 4a shows the normalized spectra of methanol solutions with different refractive indices (values measured by the Abbe refractometer, N.O.W., Tokyo, Japan). As indicated in the enlarged figure, the maximum intensities (peak values) decreased with increasing refractive indices; being 1, 0.9888, 0.9869, 0.9845, 0.9833, 0.9794, and 0.9704 for *n* = 1 (the air), 1.33 (the water), 1.3364 (*V_methnaol_*/*V_solution_* = 1/5), 1.3370, 1.3378, 1.3391, and 1.3402 (*V_methnaol_*/*V_solution_* = 1), respectively. The normalized spectra of ethanol solutions with different refractive indices are shown in Figure 4b. The enlarged figure again indicates that the peak values decreased with increasing refractive indices; being 1, 0.9878, 0.9712, 0.9639, 0.9581, 0.9550, and 0.9496 for *n* = 1 (the air), 1.33 (the water), 1.3415 (*V_ethnaol_*/*V_solution_* = 1/5), 1.3439, 1.3470, 1.3518, and 1.3589 (*V_ethnaol_*/*V_solution_* = 1), respectively.

### 3.2. Sensitivity

The sensitivity, *S*, (in RIU) of the OFS in measurements of a specific type of solution can be defined as [9]:

(4) $$S=\frac{∆T}{\left(\frac{∆A}{∆n}\right)}$$

where ∆*T* is the resolution of the spectrometer to the light source and ∆*A*/∆*n* is the slope of the normalized intensity to the refractive index. In the present setup, ∆*T* = (significant digit or analyzable value of the spectrometer to the light source)/(maximum readable value of the spectrometer to the light source) = 10/60,000~1.7 × 10^−4^. After plotting the maximum intensity against the refractive index, the slope ∆*A*/∆*n* can be derived by linearly fitting all points.

**Figure 5:**
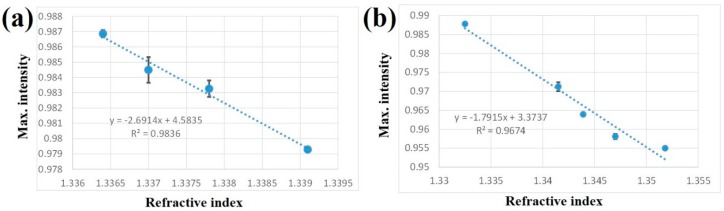
The maximum intensity (peak value) as a function of the refraction index for (**a**) methanol and (**b**) ethanol. The slope in each panel was derived by linearly fitting all data points.

For methanol sensing, the slope was 2.69 (see Figure 5a, R^2^ = 0.9836) and the calculated sensitivity was 6.3 × 10^−5^ RIU. From Equation (3), this value corresponded to a minimum detectable *V_methnaol_*/*V_solution_* of 3.6 × 10^−3^, 0.36%, or 3600 ppm. Kawano et al. developed a refractometric OFS for the assessment of methanol presence in biodiesel [13]. It exhibited a standard uncertainty of 0.6% v/v of methanol in biodiesel within the methanol concentration ranging from 0% to 25% v/v [13].

In ethanol sensing, the fitted slope was 1.79 as indicated in Figure 5b (R^2^ = 0.9674), giving a sensitivity of 9.5 × 10^−5^ RIU. The corresponding minimum detectable *V_ethnaol_*/*V_solution_* was 1.8 × 10^−3^, 0.18%, or 1800 ppm. The present OFS can be used to detect different liquors with a range of alcohol by volume (ABV). Morisawa et al. reported a novel and very simple plastic optical fiber (POF)-type sensor for detecting alcohol concentration in liquors such as beer (5% ABV) and whisky (40% ABV) in less than one minute [14]. Another OFS, based on the simultaneous immobilization of alcohol dehydrogenase and nicotinamide adenine dinucleotide cofactor (NAD+) onto optical fiber by sol–gel technique, was developed for measurements of ethanol solutions in the concentration range 2%~18% [15].

### 3.3. BSA Binding

**Figure 6:**
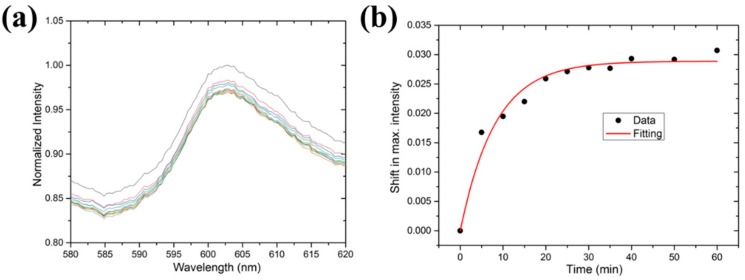
(**a**) The normalized spectra of BSA binding to the sensor surface at different time points. (**b**) The shifts in the maximum intensity at different time points. The binding kinetics were exponentially fitted to obtain the affinity.

Figure 6a shows the normalized spectra of BSA binding to the sensor surface at different time points. Although the signals were a little noisy, it was evident that the peak values decreased with increasing time points. The shifts in the maximum intensity, compared to that at time = 0, at different time points are plotted in Figure 6b. As shown, the signal saturated after about 20 min, and thus binding kinetics could be fitted to the simple Langmuir model as [16]:

(5) $$y=y_{0}\left[1-e^{-\left(k_{on}\left[c\right]+k_{off}\right)t}\right]\sim y_{0}\left[1-e^{-\left(k_{on}\left[c\right]\right)t}\right]\;.$$

In this equation, *y* is the signal, *y*_0_ is the saturated signal, [*c*] is the concentration, *k_on_* is the association rate, and *k_off_* is the dissociation rate. Since this binding is very strong and almost irreversible, *k_off_* is assumed to be 0. The fitted curve (red in Figure 6b) gave an association rate of about 6.7 × 10^4^ (Ms)^−1^. This value is close to that observed in streptavidin reacting with surface-immobilized biotin-BSA conjugates by using label-free oblique-incidence reflectivity difference (OI-RD) microscopy [16]. Therefore, the present OFS can serve as a biosensor for detecting biomolecular interactions such as antibody–antigen bindings, DNA hybridizations, and protein–carbohydrate reactions.

## 4. Conclusions

In this paper, a bare optical fiber sensor based on surface plasmon resonance was reported. Compared to other D-shaped and D-type OFSs, the sensing area of the present OFS is at one end of the fiber, making it suitable for in situ and clinical applications. This sensor was first used to measure the refractive indices of various liquids including methanol and ethanol solutions. The reported sensitivity was in the order of 10^−4^~10^−5^ RIU, comparable to that of other OFSs. Moreover, binding of proteins onto the sensor surface was monitored in real time, and the affinity could be derived from fitting the binding kinetics. By virtue of the advantages of this OFS—e.g., low cost, small size, flexibility, easy fabrication, and in situ measurement—this sensor has great potential for clinical applications such as measuring allergens, antibody–antigen interactions, and even circulating tumor cells in the blood.

## References

[1] Sun Y.S. (2014). Optical Biosensors for Label-Free Detection of Biomolecular Interactions. Instrum. Sci. Technol..

[2] Sharma A.K., Jha R., Gupta B.D. (2007). Fiber-optic sensors based on surface plasmon resonance: A comprehensive review. IEEE Sens. J..

[3] Sun Y.S., Landry J.P., Fei Y.Y., Zhu X.D., Luo J.T., Wang X.B., Lam K.S. (2008). Effect of fluorescently labeling protein probes on kinetics of protein-ligand reactions. Langmuir.

[4] Sun X.L., Shu X.W., Chen C.H. (2015). Grating surface plasmon resonance sensor: angular sensitivity, metal oxidization effect of Al-based device in optimal structure. Appl. Optics.

[5] Sun Y.S., Li C.J., Hsu J.C. (2016). Integration of Curved D-Type Optical Fiber Sensor with Microfluidic Chip. Sensors.

[6] Villuendas F., Pelayo J. (1990). Optical fibre device for chemical seming based on surface plasmon excitridon. Sens. Actuat. A.

[7] Luan N., Wang R., Lv W., Yao J. (2015). Surface plasmon resonance sensor based on D-shaped microstructured optical fiber with hollow core. Optics Express.

[8] Wang S.F., Chiu M.H., Chang R.S. (2005). New idea for a D-type optical fiber sensor based on Kretschmann’s configuration. Optics Eng..

[9] Wang S.F., Chiu M.H., Hsu J.C., Chang R.S., Wang F.T. (2005). Theoretical analysis and experimental evaluation of D-type optical fiber sensor with a thin gold film. Optics Commun..

[10] Roh S., Chung T., Lee B. (2011). Overview of the characteristics of micro- and nano-structured surface plasmon resonance sensors. Sensors.

[11] Esteban O., Diaz-Herrera N., Navarrete M.C., Gonzalez-Cano A. (2006). Surface plasmon resonance sensors based on uniform-waist tapered fibers in a reflective configuration. Appl. Optics.

[12] Bueno F.J., Esteban O., Diaz-Herrera N., Navarrete M.C., Gonzalez-Cano A. (2004). Sensing properties of asymmetric double-layer-covered tapered fibers. Appl. Optics.

[13] Kawano M.S., Kamikawachi R.C., Fabris J.L., Müller M. Fiber optic sensor for methanol quantification in biodiesel. Proceedings of the 23rd International Conference on Optical Fibre Sensors.

[14] Morisawa M., Muto S. (2012). Plastic Optical Fiber Sensing of Alcohol Concentration in Liquors. https://www.hindawi.com/journals/js/2012/709849/.

[15] Tarca R.C., Caraban A.M., Bota S., Tarca I.C., Dergez A., Cozma A.C. (2014). A New Optic Fiber Sensor for Measuring the Concentration of Ethanol in Wine. Rev. Chim-Bucharest..

[16] Sun Y.S., Landry J.P., Fei Y.Y., Zhu X.D. (2013). An Oblique-Incidence Reflectivity Difference Study of the Dependence of Probe-Target Reaction Constants on Surface Target Density Using Streptavidin-Biotin Reactions as a Model. Instrum. Sci. Technol..

